# Facilitating an early career transition pathway to community nursing: A Delphi Policy Study

**DOI:** 10.1002/nop2.355

**Published:** 2019-09-30

**Authors:** Diane Chamberlain, Clare Harvey, Desley Hegney, Lily Tsai, Sandy Mclellan, Agnieszka Sobolewska, Elspeth Wood, Joyce Hendricks, Troy Wake

**Affiliations:** ^1^ College of Nursing and Health Sciences Flinders University Adelaide SA Australia; ^2^ School of Nursing, Midwifery and Social Science Central Queensland University Townsville Qld Australia; ^3^ School of Nursing, Midwifery and Social Sciences Central Queensland University Brisbane Qld Australia; ^4^ School of Nursing University of Adelaide Adelaide SA Australia; ^5^ School of Nursing, Midwifery and Social Science Central Queensland University Mackay City Qld Australia; ^6^ School of Nursing, Midwifery and Social Science Central Queensland University Wide Bay Qld Australia; ^7^ Department of Health Mackay Health Service Mackay Qld Australia

**Keywords:** community health nursing, consensus, Delphi technique, education continuing, health transition, health workforce, model nursing, policy, safety management

## Abstract

**Aim:**

To further develop and validate a new model of the early career transition pathway in the speciality of community nursing.

**Design:**

Delphi policy approach, guided by a previous systematic review and semi‐structured interviews.

**Methods:**

Four rounds of an expert panel (*N* = 19). Rounds one, two and four were questionnaires consisting of a combination of closed (Likert response) and open‐ended questions. Round three comprised of a focus group conducted using virtual meeting technology.

**Results:**

The final model demonstrated reliable and valid measures. There were deficiencies in “pre‐entry”—where the marketing of community nursing was negligible and the support around orientation informal and minimal, mainly due to tight budgetary concerns. Community practice holds a whole new dimension for nurses transitioning from acute care as the concept of “knowing your community” took time and support—time to be accepted reciprocally and develop a sense of belonging to the community.

## 1 INTRODUCTION

Healthcare systems are undergoing substantial reform across the world in response to the needs of growing ageing population and chronic disease (World Health Organization, 2015). There has been a parallel shift and redesign of healthcare systems away from acute care and towards primary healthcare or community settings, terms which are often used contemporaneously (Barrett, Terry, Lê, & Hoang, 2016). Examples of primary health care include general practices and community health centres. Educational institutions, correctional facilities and domiciliary settings can also be sites for the provision of community health care.

Having a capable nursing workforce able to deliver proper and appropriate care in these settings is important (Smith & Herriot, 2017). A competent community nursing workforce is realized by employing new graduate nurses and by inspiriting experienced and proficient nurses to transfer from employment in acute care to community practice.

## 2 BACKGROUND

Currently, there is a meaningful body of research about career pathways, job fulfilment and retention strategies in the acute care (Moloney, Boxall, Parsons, & Cheung, 2018; Twigg & McCullough, 2014). Conversely, the research on workforce issues in the primary and community healthcare sector is limited (Humphreys et al., 2017). The changing health needs of society and the need to transfer healthcare services into the community have initiated an exponential evolution of community nursing needs both in Australia and globally (Pearson, Hegney, & Donnelly, 2000). Given the global shortage of nurses (Kingma, 2018; Marć, Bartosiewicz, Burzyńska, Chmiel, & Januszewicz, 2018), there is an obligation to retain community nursing workforce. Workplace factors play a statistically significant role in staff recruitment, job satisfaction and retention (Castaneda & Scanlan 2014). Commissioning new graduate nurses and supporting experienced nurses to transfer from acute care milieus to community practice settings can facilitate community nursing workforce development.

This study is phase three of a project, commissioned by the Queensland Government, Australia, to investigate and design a model for the early career transition pathway in the speciality of community nursing. This work builds on phase 1 comprising of a systematic review (Harvey, Hegney, Sobolewska, et al., 2019) and phase 2 consisting of a semi‐structured interview study of community nurse (Harvey, Hegney, Tsai, et al., 2019). The systematic review identified a deficiency in the published evidence, especially in the area of “pre‐entry,” a concept referring to a point in time before commencing a transition career pathway (Harvey, Hegney, Sobolewska, et al., 2019). The semi‐structured interviews from phase 2 supported a deficiency in the “pre‐entry” time point, along with highlighting weaknesses in the formal orientation process and general support when transitioning into community practice.

The aim of this study was to further develop and validate through consensus a preliminary model as a basis for the early career transition pathway in the speciality of community nursing.

The objectives were to identify and expand the knowledge concerning:

1. the entry points to community nursing practice;
2. the scope of the practitioner's community roles; and
3. the mechanisms underpinning community practice careers.

This exploration and consensus are essential to understand the inhibitors and enablers related to a community practice early career pathway. This knowledge can, in turn, inform policies that encourage a sustainable nurse workforce in the 21st century that is responsive to the healthcare needs of the community.

## 3 DESIGN

We selected a Delphi policy approach, guided by a previous systematic review (Harvey, Hegney, Sobolewska, et al., 2019) and semi‐structured interviews (Harvey, Hegney, Tsai, et al., 2019). The approach was chosen to refine, substantiate and finalize an early career pathway model for Registered Nurses in the area of community care. The essence of the Delphi policy method is to provide a factual basis for an argument for or against an issue, policy or problem (Rayens & Hahn, 2000; Turoff, 1970). We used a Lockean philosophical approach for forming consensus, based on what is known or observed from data inductively and to find agreement on issues between different individuals (Mitroff & Turoff, 2002). The Delphi technique is applied when examining an area with a scant empirical research base where there may be no definitive answers (Keeney, Hasson, & McKenna, 2001).

**Figure 1:**
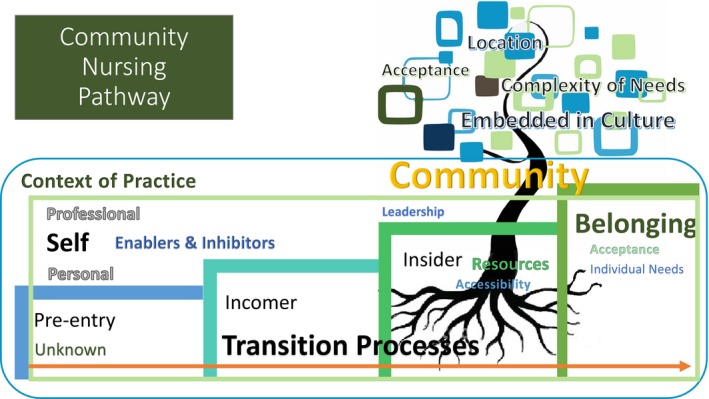
Preliminary model for the early and rapid transition pathway to specialist community nursing

The TRANSition to a Nursing SPECiality in differing contexts of practice (TRANSPEC; Chamberlain, Hegney, Harvey, Knight, & Tsai, 2019; Hegney et al., 2019) is a theoretical model developed from our previous work on early career and rapid transition to specialty practice. TRANSPEC includes the major concepts of “self;” “professional and personal,” “transition processes;” “formal and informal;” a “sense of belonging;” and the “context of practice.” Box contains the definitions of these concepts, and Figure 1 presents the preliminary model. As can be seen in Figure 1, in these concepts are three areas of transition: Pre‐entry, Incomer and Insider. These transition areas incorporate enablers and inhibitors.

Box 1 **Glossary of terms**

1 **Context of specialty**: a term used to describe the interconnected factors, experiences and opportunities which enable or inhibit a nurse as they progress across the continuum of specialty community practice. Is framed by professional and organizational elements. Organizational elements impacting on specialty nursing practice include the geographical location, size and capability of the health service, the community in which the healthcare facility is located and the diversity of health care delivered by the health service to that and other communities (COAG Health Council, 2014; National Nursing Organisations, 2004; Nursing and Midwifery Board of Australia, 2018; Queensland Health, 2016).

**Specialty nursing practice**: focuses on a distinct area of nursing activity and is based on a core body of nursing knowledge that is continually developed and refined by practice, research and innovation.

**Transitioning clinician**: Clinician transitioning into a community nursing specialty context.

**Transition processes**: Any process—formal or informal that has an impact on the clinician’s transition into the role

Sense of belonging: Feeling of acceptance and inclusion into the team, organization and speciality

**Pre‐entry**—The point in the transition before entry where there are factors which impact the clinician prior to entry into the specialty area

**Incomer**—The point in the transition where the clinician who has recently entered into the specialty area and there are factors related to this experience.

**Insider**—The point in the transition where the clinician feels a sense of belonging, being accepted, respected, included and supported as a member of the specialty team. There are factors related to this experience.

A Delphi policy approach was appropriate to this study, as the published information was particularly negligible in the Pre‐entry area of transition to community speciality nursing practice. Eliciting a divergence of opinions is pivotal in a Delphi policy approach. We chose a combination of a Delphi technique rounds and a focus group to explore divergence of opinions and the feasibility of the preliminary model. This combination also allowed for validating the credibility or internal validity of participants' views, narrations and importance ratings that were formulated through the study (de Loë, Melnychuk, Murray, & Plummer, 2016). The focus group also allowed for the enhancement of the original findings by bringing additional sources of evidence.

## 4 METHOD

### 4.1 Expert panel selection and recruitment

A regional health service office in Queensland, Australia, assisted with the identification and selection of key expert stakeholders in regional Queensland Health. As per the selection criteria, the participants were specialist community clinical nursing practitioners or nursing directors of community practice. This sample provided a blend of clinical care, expert speciality community practice and nursing policy expertise. This was a homogenous group of 19 experts who were invited and signed consent to take part.

### 4.2 Questionnaire development

The electronic questionnaires were developed using the online survey platform Survey Monkey™. The questionnaires were piloted using a separate panel of clinicians and qualitative researchers to improve usability and validity and for quality control. Wording and format changes occurred over two iterative cycles before being distributed to the expert panel.

The survey was conducted in three of four rounds from August to September 2018. The objective of the study, the questionnaire content and the scoring method were explained to the experts, who then scored each item in each question. They could also add more items or comments if they thought it appropriate, or if they wished to explain and contextualize their responses.

### 4.3 Delphi Round 1

Experts were presented with the preliminary model figure and an explanation about the model concepts. They were given a combination of open‐ended questions requiring comments and narration and closed questions where items and statements were rated for importance using a 5‐point Likert scale from 1 (less important)–5 (very important). This combination allowed panel members the freedom to comment and rate and different aspects of the preliminary model. Three reminders were sent by email to complete the questionnaires. Closed Likert items that achieved consensus were retained.

### 4.4 Delphi Round 2

Prior to the second round, the selected items, their first‐round standardized group mean ± *SD* values and the newly added views were compiled and sent confidentially to each expert by email. Round two consisted of open‐ended and closed Likert response questions and was designed using the generated items from round one.

### 4.5 Round 3 focus group

Experts gave feedback in a face‐to‐face online meeting room. Focus group technique was used to facilitate interactions. Focus groups centre on the use of interaction among participants as a way of accessing data that would not emerge if other methods were used. The data emerging from focus group interactions have a high level of face validity, because panel members confirm, reinforce or contradict the arising content (Keeney et al., 2001). The focus group was structured on knowledge gaps related to the concepts, phases, enablers and inhibitors in the transition process. A moderator led the open discussion to allow independent and novel thoughts to be gathered without limitation to any particular parts of the pathway. This open discussion explored and clarified the experts' views in ways that would be less easily accessible in a one‐to‐one interview. Similar suggestions were grouped together, where appropriate, with a group discussion to clarify and evaluate each idea. The discussion was recorded with informed consent. The resultant similar statements on concepts, phases, enablers and inhibitors informed round four.

### 4.6 Delphi Round 4

The panel was presented with an updated theoretical model informed by round one, two and three where qualitative data were transformed into quantitative question items. The panel was asked to rate items using a 5‐point Likert scale with the option of an open‐ended response to their choice. Items with a group mean score of more than three were retained. Refer to Figure 1 for the Delphi pathway.

### 4.7 Analysis

In round one, two and four, the resultant quantitative data tables generated by the electronic survey were exported to Stata 15 software for analysis. Standardized group means and standard deviations (*SD*s) were used to compare movement between Delphi rounds as a measure of both stability and convergence (Greatorex & Dexter, 2000). The group mean, as a measure of central tendency, “represents the group opinion of the panel” (Greatorex & Dexter, 2000, p. 1018). The standard deviation, as a measure of spread, “represents the amount of disagreement within the panel” (Greatorex & Dexter, 2000, p. 1018). The median is also reported for comparison to the group mean as an indicator of the direction of the group response.

Cronbach's alpha (*α*) was used during each round of the Delphi process to determine the internal consistency of survey questions or items. Cronbach's *α* was also used as a measure of homogeneity for the ratings. Increasing homogeneity was considered to be an indication of consensus among the panellists. An a priori *α* of .7–.9 was used to define consensus (Tavakol & Dennick, 2011).

The overall agreement among the experts was determined with the intra‐class correlation coefficient (ICC), with consensus and stability tested by two‐way random ANOVA with absolute agreement. ANOVA use is based on the normality of the distribution of means rather than the data. As per the Central Limit Theorem, sample sizes >5 or 10 per group, the means are approximately normally distributed regardless of the original distribution (Norman, 2010). The ICC is interpreted as follows: ≤.40, poor consistency or large variation in opinion; .41–.74, acceptable consistency; and ≥.75, good consistency (Heiko, 2012).

Qualitative data were collated from round one and two as open‐ended answers and three in the form of a transcribed audio recording of the focus group discussion provided by the participants. Data were analysed using content analysis (Burnard, 1991). First, statements were identified that were either the same or very similar. These statements were grouped together and themes developed around statements that were in the same area of interest until nothing similar emerged. These were kept as worded and included directly in round 4 as transformed quantitative statement items.

### 4.8 Ethics

The study was approved by the Central Queensland University Human Research and Ethics Committee and the Queensland Health ethical committee.

## 5 RESULTS

### 5.1 Round 1

In round 1, 12 of 19 experts completed the survey (65% response rate). Those who did not contribute were on leave or had left their positions. Panel members were recruited from community specialities and executives involved in mental health, midwifery, child, youth and family health, outreach Indigenous health, community nursing and community care for the older person. Only 20% of the panel members planned community practice as a career pathway. The qualifications of this panel focused more on their specialty of practice (i.e., mental health) rather than on community practice principles or community or primary health postgraduate education.

There were 59 items rated in the closed rating statement questions. Consensus and internal consistency between survey questions was strong except for the inhibitors in the theme of Self (Professional), in the time frame of Pre‐entry (Cronbach's *α* = .673). As this was an area deficient in published information, they were retained.

Table 1 shows the mean rating, *SD*, median and Cronbach's alpha for enablers and inhibitors of the themes of Self (Personal and Professional). As can be seen in Table 1, the highest rating item for enablers was in the concept of Self (professional at incomer) “critical thinking ability” (standardized mean = 4.50). The highest rating item for inhibitors was in the concept of Self (professional at incomer) “lack of support” (standardized mean = 4.70).

**Table 1:** Round 1 closed question ratings for the concepts of professional and personal self‐stratified by enablers and inhibitors

	Enablers				Inhibitors		
	Standardised mean	Median	*SD*		Standardised mean	Median	*SD*
**Self‐Professional** ***Cronbach's alpha*** **Pre‐entry: .926** **Incomer: .877**				**Self‐Professional** ***Cronbach's alpha*** **Pre‐entry: .673** **Incomer: .925**			
Critical thinking ability				Lack of recognition of previous knowledge and skills			
Pre‐entry	4.40	5.00	0.84	Pre‐entry	4.40	5.00	0.84
Incomer	4.50	5.00	0.85	Incomer	4.00	4.00	0.82
Teamwork ability				Lack of available positions in the program			
Pre‐entry	4.20	4.00	0.79	Pre‐entry	4.30	4.00	0.67
Incomer	4.20	4.00	0.79	Incomer	4.40	5.00	0.84
Clinical decision‐making ability				Lack of clinical placement in a specialty as an undergraduate			
Pre‐entry	4.10	4.00	0.88	Pre‐entry	3.40	3.00	1.26
Incomer	4.30	4.50	0.82	Incomer	3.50	3.50	1.08
Future aspiration in community practice				Lack of support			
Pre‐entry	4.10	4.00	0.74	Pre‐entry	—	—	—
Incomer	3.70	4.00	0.82	Incomer	4.70	5.00	0.48
Competence level [professional and clinical]				Lack of education processes			
Pre‐entry	3.90	4.00	0.99	Pre‐entry	—	—	—
Incomer	3.80	4.00	1.03	Incomer	4.20	4.00	0.79
Clinical placement in a community specialty as an undergraduate registered nurse				Poor acceptance by community [and culture]			
Pre‐entry	3.90	4.00	0.99	Pre‐entry	—	—	—
Incomer	3.00	3.00	1.33	Incomer	4.20	4.50	0.92
Knowledge level							
Pre‐entry	3.50	3.00	0.85				
Incomer	3.90	4.00	0.99				
Previous clinical experience in the speciality or similar							
Pre‐entry	3.20	3.00	0.92				
Incomer	4.00	4.00	1.25				
Leadership skills							
Pre‐entry	3.20	3.50	1.32				
Incomer	3.40	4.00	0.97				
**Self‐Personal (7)** ***Cronbach's alpha*** **Pre‐entry: .974** **Incomer: .933**				**Self‐Personal (6)** ***Cronbach's alpha*** **Pre‐entry: .905** **Incomer: .949**			
Resilience				Inadequate remuneration			
Pre‐entry	4.30	4.00	0.67	Pre‐entry	3.90	4.00	1.10
Incomer	4.10	4.50	0.99	Incomer	4.00	4.00	1.25
Positive reason for the transition				Fear of unknown or failure			
Pre‐entry	4.30	4.00	0.67	Pre‐entry	3.70	4.00	0.95
Incomer	4.20	4.00	0.63	Incomer	3.80	4.00	0.79
Motivation				Isolation from friends and family			
Pre‐entry	4.20	4.00	0.63	Pre‐entry	3.60	4.00	0.97
Incomer	4.20	4.50	0.92	Incomer	3.30	3.50	0.82
Coping ability				Anxiety			
Pre‐entry	4.20	4.00	0.63	Pre‐entry	3.40	3.00	0.84
Incomer	4.10	4.00	0.88	Incomer	3.90	4.00	0.74
Commitment level				Relocation disruptions from friends and family			
Pre‐entry	4.10	4.00	0.57	Pre‐entry	3.20	3.50	0.92
Incomer	4.10	4.00	0.88	Incomer	3.40	3.50	0.70
Self‐care quality				Work/life balance disruptions			
Pre‐entry	4.10	4.00	0.88	Pre‐entry	3.10	3.00	0.88
Incomer	4.00	4.00	0.82	Incomer	3.30	3.00	0.67
Self‐confident				Lack of respite from community			
Pre‐entry	3.80	4.00	0.79	Pre‐entry	—	—	—
Incomer	3.80	4.00	0.63	Incomer	3.50	3.50	0.85

Overall, the average agreement between panel experts was strong, ICC .939 (95% CI: 0.873–0.980), *F*(10,530) = 16.32, *p *< .0001. The total Cronbach's Alpha for round 1 model was very strong .939.

The open‐ended questions sought statements focused on enablers and inhibitors. The statements were organized into concepts of strategic measures (see Table 2); Self, both personal and professional (see Table 3); and transitioning processes (see Table 4). New concepts emerged to inform the theoretical model. These included Funding (“funding models [are] a concern” and “paid training position with opportunity to gain further permanent positions”); Supervision and Support: (“mentoring and supervision should be mandatory with adequate time allowed”); and the Workplace Environment: (“need good leaders who can manage diverse teams”).

**Table 2:** Round 1 pre‐entry narratives based on open‐ended questions about strategic measures

Strategic measures	Enablers	Inhibitors
Transition positions resourced	“Ongoing support for training courses with dedicated training courses with replacement staff available … [and] maintain these courses” (P4) “There should be identified positions that are specifically designated for new staff to develop into” (P9) “Preceptors must be trained” (P5)	“Need to [have] base grade (entry level) positions” (P5) “Absence of… careful selection of staff … and opportunities for … trial placements” (P9) “There are limited positions available” (P1)
Marketing of Community Nursing career	“Pathways into community may need to be flexible … need to match the values that are needed for the community setting … have clear pathway” (P5) “More visible career pathway” (P6) “Marketing the flexibility and autonomy of practice, that community nursing is relational and person‐centred” (P7) “Better marketing and promotion of community nursing as a speciality” (P8) “Advertising the positives” (P10) “Early career development must start early … market the work to the undergraduates” (P5) “Embedding the recognition that community pathway is valued” (P2)	
Pre‐entry placements	“Clinical placement with students [for successful recruitment]” (P1) “Providing postgraduate qualification in community speciality area” (P2) “Adequate, sufficient clinical placements during training in the community” (P11) “More clinical experience in the community sector as undergraduate. Exposure to the different types of clinical positions” (P1)	“Universities must ensure PHC concepts and placements occur in undergraduate years” (P5)
Positions available on completion of transition program		“At the moment, there's limited opportunities to progress upward” (P2) “[A need for] opportunities for career growth” (P3) “A lack of career aspirational opportunities in community practice has hindered recruitment” (P2) “There should be identified positions that are specifically designated for new staff to develop into…This will, by necessity, mean that senior staff are upskilled in clinical supervision” (P10)
Funding	“Paid training position with opportunity to gain further temporary or permanent positions” (P3)	“Funding models are a concern” (P7)

**Table 3:** Round 1 pre‐entry narratives based on open‐ended questions about the personal and professional self

The self	Enablers	Inhibitors
Personal		
Motivation & Passion	“I wanted to practice in the community as soon as the right opportunity presented itself” (P10) “Motivation is essential” (P2) “Community nursing was where I felt most aligned with my professional ideals” (P7)	
Resourcefulness	“(Own) ability to gain the child health nursing skills and learning by uni courses and student placement” (P4)	
Transition reason positive	“Having exposure to community‐based practice provides a greater understanding of roles and importance of these roles in the continuum of health care” (P2)	
Commitment high	“Sufficient hands on experience prior [to] making commitment” (P5)	
Resilience high	“Need life experiences to work in remote areas. Idealism is not an ideal pre‐requisite” (P1)	“Training courses need to develop resilience” (P2)
Self‐care quality	“You need to be able to care for yourself ‐ have good self‐care practices because often you are on your own” (P1)	“The demands placed on you can be high and client expectation can often be unrealistic” (P1)
Self‐confidence	“Person needs to be able to be confident to work independently and confidently and make decisions. Good concept of self, ability to take leadership, think critically and have a reasonable level of social intelligence will have an impact on the success of the nurse” (P4)	“While self‐confidence is desirable, [it] must not be excessive” (P2)
Professional		
Previous experience in community	“Completion of an undergraduate nursing degree is the only amount of experience that is essential to pre‐entry” (P9) “New graduates should be able to work in community with sufficient and appropriate support” (P10) “Depend[ing] on the role … some roles could take newly graduated nurses… if the model of care supported that” (P1) “Nursing degree with some experience but no set time” (P5) “Recognition of the completed course” (P4)	
Clinical decision‐making & critical thinking	“Need some good concepts in primary health care and ability to problem solve well” (P5) “Education on the complexity and challenges of the role and reward as a clinician” (P6)	“Many of the community nursing/ roles are largely autonomous … need 3 years (of) experience” (P1) “At least 1 year to enable isolated practice and clinical decision‐making with remote supervision” (P7)
Teamwork ability	“Ideally, you would start with a team with more experience practitioners … recognition that community‐based nursing services are valued members of the health care team” (P2) “Sufficient staff on site for both staff safety and collaboration” (P4) “Digital clinical decision‐making tools [may be useful]” (P7) “Team connection/network and community of practice options” (P7)	“Difficult to feel part of a team when your practice is in isolation. Take effort to be part of a team” (P1) “Team structures provide clinical governance that ensures patient safety. An inability to function as a cohesive team member would compromise the nurses” practice” (P5)
Competence, knowledge & skills—generalist	“Sufficient hands on experience prior to making commitment … realistic onsite experience as graduate” (P4) “Broad experience across generalist nursing to have capacity to manage both specific conditions … [and] comorbidities” (P8) “Skills and knowledge based on sole practitioner … ensuring nurses receive skills and knowledge required to fulfil their role” (P2)	“At least 2 years post‐graduation to consolidate learning. Level of experience would depend on position” (P2) “1–2 years post graduate (experience) before embarking on rural placement then supervision is required” (P4) “Nurses concerned about losing acute care clinical skills” (P2)
Clinical placement to understand community practice principles	“Rotational experience programs when working within the sector” (P1) “Gain the child health nursing skills and learning by courses and student placement” (P3) “Lots of opportunities to do different parts of community work” (P5)	
Leadership for autonomous practice		“Many of the community nursing roles are largely autonomous therefore need 3 years' experience. However, some roles could take newly graduated nurses if the model of care supported that” (P2)
Professional maturity		“Recruitment strategies need to match the values that are needed for the community setting and to work out if the candidates are good at problem solving and working independently or ability to work toward this” (P6) “The recruitment process itself needs to be transparent and robust… Questions need to be able to identify potential candidates who are flexible in their thinking, mature and robust enough to practice with developing autonomy” (P10)
Self‐reflection regarding scope of practice	“I knew after completing a community placement that community nursing was where I felt most aligned with my professional ideals as an entry nurse and it made the most sense. I completed midwifery studies three years later and again the model of health and wellness aligned with my values and ideals as a nurse” (P7)	
Mentors/ Preceptors/Supervision	“Preceptor on board then supervision and mentoring models within the community organisation” (P7) “Appropriate supervision of staff … preceptorship and mentorship … to challenge and grow” (P1) “Support and mentoring from experienced RN/CN/CNC” (P6) “Role model who can provide coaching and mentoring” (P8) “Mentoring and supervision should be mandatory, with adequate time allowed” (P9) “Clinical supervision and support and timely access to support for decision‐making inputs” (P7)	

**Table 4:** Round 1 transitioning processes concept for the incomer category narratives based on open‐ended questions focused on strategic measures

Strategic measures	Enablers	Inhibitors
Transition programs & Orientation	“Post graduate specialisation program … rotational programs between similar streams (e.g. paediatrics and child health)” (P1) “Effective orientation” (P8)	“Individual learning and a transition support program … [should] matches or articulates with universities … contribute to a career in the community” (P5)
Buddy system	“Need peer support network” (P3)	
Time frame tailored to individual needs	“Transition support needs to last beyond the first year” (P6) “[Have] clearly identified professional development/educational goals” (P10) “[Availability of] adequate relief time after stressful incidents” (P1)	“There may be the requirement for additional skills however we fail to recognise that a lot of skills are transferrable” (P1)
Person, family & community centred assessment skills	“A true sense of holistic care ‐ need to see the person as a whole and part of the community, to think about the needs of our patient beyond the hospital” (P2) “Qualifications are required in area of work to ensure good understanding of what is happening to/for the client, to be able to conduct appropriate assessments and develop appropriate plan of action and evaluation. Having a holistic approach is important, understanding what factors are impacting your client or their condition” (P3) “Ongoing study in theories and models of primary health care and patient centred care” (P7)	“Focus on specialty areas in tertiary level training…required for remote area nursing” (P2)
Knowledge of community culture	“Connection to space or country” (P4) “Primary source of contact for some communities” (P3) “Site specific specialities especially in diverse roles e.g. Indigenous communities/graziers/station settings, rural townships” (P1)	“Community nursing in remote areas is not for faint hearted…persons should be aware of issues prior to accepting positions… isolation etc” (P2) “Need generalist training in rural areas including cultural awareness/safety/driving skills” (P2)
Knowledge of referral pathways	“[The community nurse is a] repository of knowledge of where to go or how to solve a problem (health or lifestyle)” (P3)	
Practice in others personal space rather than hospital space	“Capacity to work both with hospital specialists, general practitioners, and within multidisciplinary team” (P8)	
Understanding role in a multidisciplinary service	“At least 3 years of nursing in variety of different roles … outside of the acute setting” (P6) “Multidisciplinary practices where all teams work collaboratively with defined roles” (P3) “Provide experience in working across community setting” (P8) “Recognition that community‐based nursing services are valued members of the health care team and are an important component of ensuring healthy people/community” (P3)	“The value of community‐based nursing practice is under‐recognised” (P1)
Peer and community health Support systems	“Have a support network for community nurses including mentoring, clinical skills feedback/supervision, peer support network” (P2) “Support is absolute necessity … needs to last beyond first year” (P5) “[Having the] ability to recognise need for support/self‐caring strategies in dealing with difficult situations” (P1)	
Clinical skills to match community needs	“[Community nurses need to] have ability to work across both community and acute care … role of health promotion and primary health care” (P2) “[Need] a diploma in child health” (P3) “Digital clinical decision‐making tools” (P8) “Clinical decision making is also very important ‐ you are assessing your client and making decisions as to plan of care. You also need the clinical knowledge to know when this is beyond your scope of practice and need to refer on” (P2)	“In rural areas, nurses are often de facto allied health and this leads to resentment on return to city areas” (P4) “Health practices change” (P2)
Continuing education and lifelong learning support	“Have a career framework for nursing … include career pathway … provide opportunities for professional Development in the area of community‐based nursing service … include updating on evidence based best practice” (P2) “Ongoing study [is necessary]” (P6) “Support for postgraduate pathways through scholarship programs” (P1) “Ongoing professional development” (P7) “Clearly identified professional development/educational goals … and access to appropriate postgraduate education” (P9)	
Workplace environment	“Workplaces need to understand concepts such as different generations … [need] good leaders who can manage diverse teams” (P5) “Strong and competent leadership” (P8) “Ensuring nurses receive skills and knowledge required to fulfil their role” (P3) “Flexible working conditions, opportunities for career growth” (P4) “Workplaces that are happy, provide clinical supervision and are fun, interesting and engaging” (P6)	“At present there is limited opportunity to progress upward” (P3) “Higher level positions are scarce” (P1) “Lack of recognition of previous knowledge and skills greatly inhibits professional concept of self” (P1) “At present, most opportunities are in the acute care setting” (P2)

### 5.2 Round 2

Round 2 consisted of 13 open‐ended questions and 18 closed importance rating questions of 79 items. The open‐ended questions sourced statements of enablers and inhibitors that were organized into concepts of Self, both personal and professional and transitioning processes. Tables 5 and 6 show the lists of items, and Tables 7 and 8 present their associated narratives.

**Table 5:** Open‐ended questions exploring the concept of self, stratified by enablers and inhibitors

Self	Enablers	Inhibitors
Professional (incomer)	Clinical decision‐making and reasoning Problem‐solving skills Ability to ask guidance/seek out information as required Self‐confidence Self‐motivated, listen and adapt Self‐reflection Communication skills Clear professional boundaries Clear scope of practice Knowledge Resilience Organizational skills Clinical competency Being proactive/self‐motivator Critical thinking Situation awareness Sense of professional self—need to be fostered through good role model and feedback Fundamental professional knowledge Support of experienced and skilled CNCs Open to observing and learning from other work practices Innovative Practical compassionate emotional intelligence Influencer Defined career path Use informal networks	Lack of constructive feedback and guidance Recognition of burnout and compassion fatigue
Personal (incomer) Choice of transition	Connected to team	Isolation
	Explore self‐values and beliefs Cultural safety Cultural choice Good leadership and vision of community managers Opportunity for clinical supervision Ability to work autonomously Ability to manage work flow Clear definition of community roles	Perceived myth and reality from consumers and staff, and social media

**Table 6:** Open‐ended questions exploring concepts of transition processes, insider and belonging stratified by enablers and inhibitors

Concept	Enabler	Inhibitor
Transition processes		
Orientation—context	General orientation Overview and purpose of the centre Defined role, duties, policies and procedures Worked with someone for few days Community orientation—health team, health facility Referral processes Regular contact with support network Mentored by clinician in community Role evolved Broad generalist experience Networking Peer processes Coaching by existing staff	Trial and error—No one with previous experience Self‐guided and explored—available resources, network, peer, operational manuals and policies Orientation to health, driving/surviving skills Negotiating skills Following a nurse for few days is not orientation
Ideal orientation program	Supernumerary for 1–2 weeks Mentorship Regular meeting to review practice and debrief, and post orientation Formal process of supervision Allocated preceptor Recognition within educational frameworks Mediation process Community process Buddy Performance agreement Introduction to other health professionals, key stakeholders outside practice but in the area Referral processes Support networks Introduction to community Logistical orientation (e.g. time sheet, payroll) Shadow another clinician Technological programs and devices Review of local needs Self‐awareness/personal health courses	
Role of mentor	Introduction to: role; boundaries; referral pathways; local community and culture; system; culture norm Encourage progression of practice and experience Available for debrief Provide support beyond incomer period (i.e. over 1–2 years); constructive feedback; coaching; role modelling; resource guidance; structured program of regular contact; support tailored to the need of nurse Identify strength and clinical knowledge and skill gaps Assist to develop confidence and abilities Guide clinical practice reflection Assist in developing critical thinking and problem‐solving skills Available via electronic media/phone	
Amount of supernumerary experience	Depend on the role, prior experience, level of autonomy A week for experienced 2–3 weeks for inexperienced, or complex role Need guided support Senior/expert clinician in community may provide guidance to gain skills as well Up to one month in single/small remote community	Need to build into work programme Not all organisations can afford supernumerary experience Private employees may not have capacity to support supernumerary
Strategies needed	Effective mentorship (can be remote) Clear: referral pathways; orientation processes; policy; education framework; succession planning; scope of practice; professional boundaries and relational care Introduction to local community and culture Peer support Facilitate handover process Support with client care Consumer feedback Self‐care Planned program for upskilling Coaching Community of practice Receive required training early Supported supernumerary practice Tailored learning packages Study days at intervals (ideally 6 months) Health workplace Good leadership Understand different generational needs and drives Allocated lunch breaks Resourced with equipment required for the role Team to belong to Allocated space to sit Educational and personal development Linking to nursing groups Good teleconference access	
Early career entry	Undergraduate placement/exposure Effective mentorship—to support autonomous decision‐making Referral pathways Introduction to local community and culture Practice guidelines Case reviews Peer support Resilience Debriefing Self‐reflection guidelines Develop pathway for continuity of care Preceptor—have time Regular clinical supervision Good orientation Coaching Career pathway—postgraduate pathway to specialisation Professional development Structured orientation program with core competency development Good leadership and management Role expectations Positive feedback Suitability of a person for proposed role Self‐motivation Resources—nurse educators, CNCs, clinical practice facilitators Transition learning package Regular study days and placements Opportunity for work experience in different setting—that is acute care facility Clinical decision‐making tools/pathways Services can “grow our own” and bind staff long enough to gain their loyalty Provide a variety of different work to keep staff engaged and interested Flexible work conditions must be balanced with providing the services Workloads—realistic, prevent burnout Being validated for role and acknowledged Respect from wider staff for input Inclusion in planning and whole case of patient Interpersonal communication skills Computer literacy	Feeling of isolation in practice Work/life balance Lack of life experience Lack of computer literacy Volume of work involved in transition program is too large [for new graduates] Programs that do not interact are time wasters Lack of placement opportunities in undergraduate Many placements are unsuitable [for new graduates]
Insider		
Continuous Professional Development (CPD)	Able to access PD leave Learning is always ongoing and obtained through a healthy workplace Encouragement to know what opportunities are available outside the workplace Engagement in a professional association that reach out to new graduates Individual source appropriate CPD according to needs Support for CPD is essential in remote areas [but] requiring replacement staff to attend	CPD is not a formal part of my role Self‐guide [I] actively finding opportunities for CPD & trying to fit it into current workload (no relief) No formal requirements [in my role] other than … AHPRA commitment Need to identify ways of CPD Ability [for organisation] to support financially depends on staffing level Time out to travel to regional centres adds to burden of role
Strategies to retain community specialists	Research and reporting Visibility of service—build services and relationships Consumer engagement Clinical supervision and feedback Professional development supported and encouraged Teamwork and culture fit Recognition and development opportunities—that is work across both community and tertiary settings Remuneration Coaching and development conversations Good leaders Inclusive workplaces University programs with clear pathways	
Speciality specific	Approach the support differently—for example mentor/supervision may occur through video/phone Generalist training [in rural and remote community nursing] Sole practitioners—clinical practice network Supervision strategies require community involvement Innovative independent in practice	Common to both [metropolitan and rural and remote] to inform needs and services
Belonging	Effective patient advocate Sense of community belong Life experience Empathy Ability to motivate	

**Table 7:** Open‐ended questions with narration by the concept of self

Self	Enablers	Inhibitors
Professional (incomer)	“Clinical decision making and reasoning. Good at problem solving. Not afraid to ask guidance, self‐confidence” “Self‐motivated, listen and adapt. Analytic problem solving, self‐reflection, communication, listening skills, team building, leadership skills, professional boundaries, strong scope of practice, knowledge, resilience” “Organisational skills” “Clinical competency with capacity to take initiative to ask for help/seek out information as required” “Competent clinical practice, critical thinking and problem‐solving skills … being proactive” “Situational awareness” “A sense of professional self needs to be fostered which includes have good role models and good feedback and the right fundamentals of professional knowledge … support of a skilled CNC who has knowledge in the area” “Open to observing and learning from other work practices, being innovative … practical compassionate emotional intelligence to relate to both clients and staff … influencer” “Defined career path … self‐motivator” “Use of informal networks.”	“Lack of constructive feedback and guidance” “Recognition of burnout and compassion fatigue”
Personal (incomer) Choice of transition	“Important to connect to a team”	“Dealing with isolation is important”
	“Explore self‐values and beliefs, cultural safety and cultural choice” “Good leadership and vision of community managers … Opportunity for performance feedback, clinical supervision, ability to work autonomously and manage work flow” “Define community roles”	“Preconceived myth and reality from consumers and staff, and social media”

**Table 8:** Open‐ended questions with narration by concepts of transition processes, insider and belonging stratified by enablers and inhibitors

Concept	Enablers	Inhibitors
Transition processes (incomer)		
Orientation	“[I had] general orientation” “[I had] overview of the Child Health Centre, purpose of the centre, explanation of my role, duties, policies, and procedures. Worked with someone for a few days … oriented to the community, health team, and health facility … referral processes … had regular contact with support network” “I worked with one other nurse … [for] some days” “[I was] mentored by clinician in community” “[My] role evolved … [I] had broad generalist experience”	“[I obtained orientation knowledge by] trial and error … we had no one with previous experience” “Networking, exploring available resources … and peer processes” “Coaching by existing staff” “Experienced peers, operational manuals and policies” “Need orientation to health, driving/survival skills, negotiating skills” “Following a nurse around for few weeks is not orientation”
Ideal orientation program	“Supernumerary for 1–2 weeks, mentorship, regular meeting to review practice and debrief” “Formal process of supervision, allocated preceptor and mentorship, recognition within educational frameworks, mediation process” “General orientation of community process followed by unit orientation. Preceptor or buddy for initial period, performance agreement” “Introduction to other health professionals in the area … referral processes, introduction to key stakeholders outside of practice, support networks, community … time sheet, payroll … shadow another clinician … technological programs and devise” “Regular meeting post orientation” “Review of local needs … self‐awareness/personal health courses”	
Role of mentor	“Introduction into the role … boundaries and referral pathways … encourage progression of practice and experience … available for debrief … local community and culture” “Past the incomer period” “Navigate the system and culture” “Identify strength and clinical knowledge and skill gaps … provide constructive feedback and support … assist incomer to develop confidence and abilities … guide the incomer in clinical practice reflection, assist in developing critical thinking and problem‐solving skills” “Coaching, role modelling, resource guidance” “Provides structured program of regular contact with incomer, willing to work with incomer over 1–2 years … available via electronic media/phone … support tailored to the need of the nurse”	
Amount of supernumerary experience	“Depend on the role & prior experience. At least a week for experienced, 2–3 weeks for inexperienced” “Depend on … the level of autonomy” “More [than a week] in complex role … it is important to have more time” “Ideally … at least 2 weeks minimum … but guided support” “If it is limited, senior/expert clinician in community will provide guidance to gain skills” “Up to one month in single/small remote communities”	“Not always possible, needs to build into work program, not all organisations can afford supernumerary experience” “Need to understand the capacity of private employee to support supernumerary time”
Strategies needed	“Effective mentorship, clear and effective and accepting referral pathways. Introduction to local community and culture” “Clear orientation processes, policy, education framework, succession planning and peer support … scope of practice, review facilitate handover process, support with client care, consumer feedback, self‐care boundaries and review” “Planned program for upskilling” “Coaching, communities of practice” “Receive required training early” “Professional boundaries and relational care” “supported supernumerary practice, education on professional boundaries and relational care” “remote mentorship … tailored learning packages, study days at intervals … ideal for about 6 months … healthy work place … good leadership … understand different generational needs and drivers” “ensuring lunch breaks are allocated … resourced with equipment required for the role, team to belong to, allocated space to sit” “educational and personal development… linking in with nursing groups … good teleconference access”	
Early career entry	“Undergraduate experience/exposure, effective mentorship, clear and effective referral pathways, introduction to local community and culture” “Develop supported practice guidelines, case reviews, peer support, resilience, debriefing, self‐reflection guidelines, develop pathways for continuity of care, preceptor” “Regular clinical supervision” “Good orientation, coaching” “Clear career pathway, mentor, professional development” “Structured orientation program with core competency development” “Good leadership and management, clarity of processes and role expectations, positive feedback, ongoing education and training” “Suitability for proposed role … self‐motivation to improve nursing skills demonstrated” “Peer support for extended transition programs. Need a workplace that is accepting, where preceptors have time, resources such as nurse educators, CNCs and clinical practice facilitators, transition learning package … regular study days and placements” “Postgraduate pathway to specialisation” “Opportunity for work experience in acute care facility” “Mentor to support autonomous decision making, clinical decision‐making tools/pathways” “Services can ‘grow our own’ and bind staff long enough to gain their loyalty … provide a variety of different work to keep staff engaged and interested … flexible work conditions must be balanced with providing the services. Workloads need to be realistic, prevent burnout” “Being validated for role and acknowledged, respect from wider staff for input. Inclusion in planning and whole care of patient.” “[Need] interpersonal communication skills”	“feeling of isolation in practice” “work/life balance” “computer literacy” “need life experience” “the volume of work involved in transition program is too large [for new graduates]” “programs that do not interact are time wasters” “lack of placement opportunities in undergraduate is a concern however many placements are unsuitable to give outline of possible career choices”
Insider		
Continuous Professional Development (CPD)	“I am able to access PD leave.” “Learning is always ongoing and obtained through a healthy workplace that knows how to develop high performance staff and keep us engaged and learning … encouragement to know what opportunities are available outside the workplace … engagement in a professional association … that reach out to new graduates” “It is expected that the individual source appropriate CPD according to needs” “Support for CPD is essential in remote areas requiring replacement staff to attend”	“No [CPD is not a formal part of my role]. I self‐guide [to maintain it]. [I] actively find opportunities for CPD and try to fit them into current workload” “No formal requirements [in my role] other than … AHPRA commitment” “I need to identify ways.” “Ability [for organisation] to support financially depends on staffing level” “Time out to travel to regional centres adds to burden of role”
Strategies to retain community specialists	“Research and reporting, visibility of service to further build services and relationships, consumer engagement.” “Clinical supervision or feedback community with area of practice. Professional development supported and encouraged. Teamwork and culture fit” “Recognition and development opportunities, remuneration” “Opportunities to work across both community and tertiary settings” “Coaching and development conversations … good leaders, inclusive workplaces, university programs that have clear pathways”	
Speciality specific	“You may need to approach the support differently (e.g. mentor/supervision may occur through video/phone)” “More generalist training [in rural and remote community nursing]” “Nurses are often sole practitioners … clinical practice network is [important]” “Supervision strategies require community involvement and remote options” “Need to become more innovate and independent in practice”	“Research is common to both to inform needs and services”
Belonging	“Effective patient advocate … sense of community belonging” “Life experience, empathy, ability to motivate”	

New concepts from round 2 were included. One of them, as presented in Table 7, was the *choice of transition* in Self narrated as follows: (“[professional needed to] define community roles”) and (“[personal needed to] explore self‐values and beliefs, cultural safety and cultural choice”). Another new concept was the amount *of supernumerary experience* in transitioning processes. This concept is presented in Table 8 as follows: (“more [than one week] in a complex role.. important to have more time”) and *early career entry*: (“undergraduate experience/exposure, effective mentorship, clear and effective referral pathways, introduction to local community and culture”). The new concepts were added to the model and transition pathway.

There were 79 items rated in the closed rating statement questions. Consensus and internal consistency between survey statements was strong. The highest rating item for Self (professional) enabler was “satisfactory critical thinking ability” (standardized mean = 4.78) and for transitioning processes enabler “supportive staff and feeling as part of the team” (standardized mean = 4.78) and Transition processes inhibitor “lack of support” (standardized mean = 4.89). The mean rating, *SD*, median and Cronbach's alpha for enablers and inhibitors of the themes of Self (Personal and professional) are presented in Table 9; and for transitioning processes (formal and informal) and Belonging in Table 10.

**Table 9:** Closed rating question items in the concept of self stratified by enablers and inhibitors

	Enablers				Inhibitors		
	Standardised mean	Median	*SD*		Standardised mean	Median	*SD*
Self—Professional (Incomer) Cronbach's alpha: .930				Self—Professional (incomer) Cronbach's alpha: .955			
Resilience	4.67	5.00	0.50	Isolation from friends and family	4.22	4.00	0.83
Motivation	4.67	5.00	0.50	Inadequate remuneration	4.22	4.00	0.67
Self‐care quality	4.44	5.00	0.73	Work/life balance disruptions	4.11	4.00	0.93
Coping ability	4.44	5.00	0.73	Fear of failure	4.00	4.00	0.71
Commitment level	4.22	4.00	0.44	Relocation from friends and family	4.00	4.00	0.87
Positive reason for the transition	4.11	4.00	0.33	Anxiety	3.89	4.00	0.60
Self‐confidence	4.00	4.00	0.71				
Self—Professional (insider) Cronbach's alpha: .972				Self—Professional (insider) Cronbach's alpha: .797			
Critical thinking ability satisfactory	4.78	5.00	0.44	Scope of practice is outside the scope of current competence	4.67	5.00	0.50
Feel as part of the team	4.56	5.00	0.53	Lack of recognition of current knowledge and skills	4.11	4.00	0.60
Competence level recognised	4.56	5.00	0.53	Scope of practice is unpredictable	3.78	4.00	0.97
Clinical decision‐making ability satisfactory	4.56	5.00	0.73				
Recognition of current knowledge and skills by others	4.33	4.00	0.71				
Knowledge level satisfactory	4.33	4.00	0.50				
Leadership skills emerging	4.22	4.00	0.67				
Future career aspirations drive performance	4.22	4.00	0.67				
Self—Personal (insider) Cronbach's alpha: .973				Self—Personal (insider) Cronbach's alpha: .822			
Resilience level satisfactory	4.44	4.00	0.53	Fear of failure	4.33	4.00	0.50
Coping ability satisfactory	4.44	4.00	0.53	Anxiety (affects performance and relationships)	4.22	4.00	0.67
Self‐care quality satisfactory	4.33	4.00	0.71	Work/life balance disruptions	4.00	4.00	1.00
Motivation is satisfactory	4.22	4.00	0.44	Inadequate remuneration	4.00	4.00	0.50
Self‐confidence satisfactory	4.11	4.00	0.60				
Commitment level satisfactory	4.11	4.00	0.33				
Resources for support are adequate	4.78	5.00	0.44	Lack of available positions	4.44	5.00	0.73
Effective orientation	4.67	5.00	0.50	Limited feedback from others	4.44	4.00	0.53
Appropriate level of content	4.67	5.00	0.50	Program under‐resourced	4.33	5.00	1.12
Mentors effective	4.67	5.00	0.50	Too technical	4.22	4.00	0.83
Preceptors effective	4.67	5.00	0.50	Information Technology demands too difficult	4.11	4.00	0.60
Preparation program embedded in the reality of practice	4.56	5.00	0.53	High volume of information	4.11	4.00	0.78
Recognition of prior learning is respected	4.44	4.00	0.53	Overwhelming content	4.00	4.00	0.87
Time allowance for transition is adequate	4.33	4.00	0.50	No clinical placement in the specialty as an undergraduate	3.56	4.00	1.13
Supernumerary time adequate	4.22	4.00	0.97				
Clinical placement in the specialty as an undergraduate	4.00	4.00	1.00				

**Table 10:** Closed rating question items in the concepts of transition processes and belonging stratified by enablers and inhibitors

	Enablers				Inhibitors		
	Standardised mean	Median	*SD*		Standardised mean	Median	*SD*
Transition processes—formal (incomer) Cronbach's alpha: .978				Transition processes—formal (incomer) Cronbach's alpha: .937			
Supervision appropriate	4.78	5.00	0.44	Insufficient orientation	4.67	5.00	0.71
Resources for support are adequate	4.78	5.00	0.44	Lack of available positions	4.44	5.00	0.73
Effective orientation	4.67	5.00	0.50	Limited feedback from others	4.44	4.00	0.53
Appropriate level of content	4.67	5.00	0.50	Program under‐resourced	4.33	5.00	1.12
Mentors effective	4.67	5.00	0.50	Too technical	4.22	4.00	0.83
Preceptors effective	4.67	5.00	0.50	Information Technology demands too difficult	4.11	4.00	0.60
Preparation program embedded in the reality of practice	4.56	5.00	0.53	High volume of information	4.11	4.00	0.78
Recognition of prior learning is respected	4.44	4.00	0.53	Overwhelming content	4.00	4.00	0.87
Time allowance for transition is adequate	4.33	4.00	0.50	No clinical placement in the specialty as an undergraduate	3.56	4.00	1.13
Supernumerary time adequate	4.22	4.00	0.97				
Clinical placement in the specialty as an undergraduate	4.00	4.00	1.00				
Transition processes—informal (incomer) (6) Cronbach's alpha: .962				Transition processes—informal (incomer) (4) Cronbach's alpha: .899			
Supportive staff	4.78	5.00	0.44	Lack of support	4.89	5.00	0.33
Part of the team (feeling and treated as)	4.78	5.00	0.44	Work allocation	4.44	4.00	0.53
Strong role models	4.56	5.00	0.53	Conflicting information	4.44	4.00	0.53
Spontaneous effective teaching	4.44	4.00	0.53	Level of responsibility	4.33	4.00	0.50
Context of the specialty	4.44	5.00	0.73				
Culture of the specialty	4.33	4.00	0.50				
Belonging Cronbach's alpha: 1.057				Belonging Cronbach's alpha: .944			
[Positive] employer support	4.67	5.00	0.50	Workload overwhelming	4.67	5.00	0.71
Accepted (by community)	4.67	5.00	0.50	Culture of community [not included]	4.56	5.00	0.53
Supported (by specialty work colleagues)	4.67	5.00	0.50	Availability of positions post transition	4.33	4.00	0.71
Position description that is supportive of education and a learning environment	4.56	5.00	0.53	Level of responsibility overwhelming	4.33	4.00	0.71
[Positive] culture of the organisation	4.56	5.00	0.53				
Respected (by the specialty work colleagues)	4.56	5.00	0.53				
Included (by specialty work colleagues)	4.56	5.00	0.53				
Accepted (by specialty work colleagues)	4.56	5.00	0.53				
Appropriate skill mix [perception of]	4.44	4.00	0.53				
Role adequately funded	4.33	4.00	0.71				
A good fit for the community culture	4.33	4.00	0.50				

Overall, the consistency and average agreement between panel experts was strong, ICC .959 (95% CI: 0.909–0.989), *F*(8, 624) = 24.374, *p *< .0001. The total Cronbach's Alpha for round 2 model was very strong .959, higher than round 1 and demonstrating strong reliability.

### 5.3 Round 3

The focus group had nine participating experts and was 2 hr long. Additional themes and sub‐themes were included in the model that emerged through the focus group. The new themes included “safety of self, clinicians and patients,” “marketing,” “scope of practice and time” and “professional development and life‐long learning.” Table 11 documents the focus group findings.

**Table 11:** Findings from Focus Group

Themes	Sub‐themes	Narration
Safety of self, clinicians and patients	Prevention of hospital admissions	People in their own environment that's close at home rather than in an environment that's comfortable to work We look at the longer we can keep somebody at home safely To keep them safe and independent for as long as possible at home
	Social aspects in safety	It's having equity and access to services It's often soft things where damage occurs in terms of psychosocial risk that is less measurable Psychological safety is a big one, it gets tough, it gets really tough and burn out big time [Importance of] self‐care They deserve health care and that it shouldn't be that they have to travel you know exorbitant kilometres just to get basic health care [I felt] psychologically at risk a number of times in my role in community You know how to fix wounds and you know [how to] patch them up. But those social issues, if you're not aware of what's available on how to support that family further, then you know you're sending them back into another situation, the same situation
	Having insight into self and reflective practice	Need to prepare them around what looks to be fairly glamorous in terms of autonomy and the other side of the coin of autonomy is having to be the sort of person who can make decisions sometimes with limited resources and having to problem‐solve They don't know what they don't know Having that self‐awareness You know I need training in this particular area and it's that insight again of knowing where my skills and abilities are Clinical practice assessment tools Bit of self‐reflection time Know what's my capacity in my caseload You know if you're going home every night wondering if you done the right things [for] your patient safety … I don't think those feelings ever leave you … professional safety … believe in yourself
Marketing		How do we promote ourselves, can I say we don't do a good job of it
	Value in being in homes and streets	Tailor your care according to where that person's at Principals of primary health care really embodies what the essence of community is for myself Essence of community practice is very much embedded in the principles of primary health care [Having] soft skills in communication We're out there where people are on the streets in their homes with their family [The role of community nursing] is so diverse, really diverse The process of family partnership is about introducing yourself and working out what the goals are Community health roles are very important and very valid Primary health care model … preventative care … chronic conditions or short‐term conditions like whether they've comfortable with the post‐acute care … continue to rehabilitate We're very much working together with the client and having them at the centre of that care Capturing where the gaps are You take initiative, you see what the client's needs are You carry that patient with you on the journey … often relationships that you have for a number of years Advocating and championing for the services that your client needs
	Nobody knows what community nurses do—invisibility	Community looks very different to the style of nursing that you see in the acute sector and so the values Being invisible means that we get undervalued We're not visible, they can't see what we're doing so they can't know unless they actually walk in our shoes Strong focus on the primary health care principles and the foundations
	Difficult to market prevention	Preventive health care… can be complex at times because people don't always well identify their needs We're talking about prevention … most difficult things to market People aren't always thinking of the cost of preventing disease, initially they're thinking of the cost of treating the disease Trying to encourage clients to accept some services to help them maintain their independence Unless we can demonstrate measurable outcomes and our KPIs to show that yes our different input into clients here has produced something at the end, that it's really hard to attract funding or support for those roles
	Acute nurses have limited understanding of community	Prevent and promote health care in the ED, I was on the wrong end of the scale There's a whole lot of different digital platforms in every single Hospital and Health Service… so they miss people because they don't see them on their system Tried to poach or coach people across from acute wards … look like they might feel fit the model of community quite well
	Student placements essential	If they don't know what's out there and what's expected, it's bit hard to make a career choice in that direction Hard to get some placements Getting students out and giving them a decent amount of time in the community I think there is the opportunity for a post‐grad, I think it would actually be very worthwhile Good amount of practical placement in that field Clinical placements in different community settings might help
	Career pathway missing in community	There's not a lot of career pathway I think we need career days that really promote not just a hospital. We need to promote all those roles that nurses have out in the community and the importance of them It tends to be tied in with other career pathways, because it's so specialized I haven't come from a direct entry level
	Role exchange with acute RN & community RN	I don't know what I'm doing, I feel as though I need to go into acute care to consolidate my learning Ability for acute nurses to work in the community
Enablers entering community	Preceptorship AND mentorship, having a safe go‐to person	I'm sure, sometimes undergraduates could see that you're having a very nice conversation with family but not picking out the nuances of how you know your probing and you are asking the right questions We're so experienced, we forget that the nuts and bolts and having to point that out to some preceptors The ability to talk through case studies It's keeping them there, and I think it's keeping them there by providing the debrief sessions I have somebody I can contact and talk the situation through with Enabler is having a safe go to kind of clinical practice supervision Just when you're thrown out on your own, I think you really need to be able to have that contact Not common in there for nurses to get clinical supervision Clinical supervision and the tele mentoring Knowing who is it that I can go to, who is my network, who can I talk to, particularly in most isolated areas Role modelling … I observed people having some wonderful discussions and seeing how they work around with families How to support new learners Mentoring is a really big part of precepting and I'm actually precepting somebody now and as we speak and she's been with me for three weeks and I'm quite protective of ensuring that she feels supported and that she's not given any task that is beyond her ability to manage yet until she's aware of all the systems and or checking in you know how do you feel about doing that Prioritize what you feel that they're going to need first so that they're not going to sink. They can at least swim a little bit in some of those preliminary roles. And it's as sequential and gradual in terms of tasks responsibilities and taking them on board. So not setting people up to add something that's too complex to manage. If you're all brand‐new to the service … you would be looking at tasks that you'd have to be able to be with someone that can assist with that, and then gauge what tasks would be appropriate to allocate
	Generalist background	The community nurses to have that time in a generalist setting I think would be very useful Allowing clients to be the decision‐maker in their own lives without taking over their lives and being paternalistic People early in their career may already have very advanced skills … have got transferable skills
	Resilience	Resilience is a really big thing
	Helps to have a background in the bush	I would think they would really struggle because you know, you might come in with the world of knowledge and expertise, but if you're coming into an area that you know nothing about then you know you're just going to struggle, because you don't know what the social issues are and you know you're working in community so it's usually around social things that aren't working for people particularly around the health
	Attendance at conferences and PD	You don't know that the wheels moved on unless you keep engaged in your profession in some way Always making sure that you keep connected with. You know where the world closing on some sort of education process and going to the key conferences that are really important to you depending on the trends
	Clear referral pathway	it's important to have very clear referral pathways. If you are out in the community you need to know where, who to contact, how to get there, how to get your clients the help they need Referral pathways because GPS aren't even aware what we're trying to do at the moment it's word of mouth through patients, family and community
	Resourcefulness	Resourcefulness I think it's a really big one when you go out into the community There are some clinicians who are in community that don't have IMR access … This is clinical risk for those clients that have been transferred across You can teach people and educate them till the cows come home… [if] they don't have basic resources you know because of poverty or life situations then they're never going to be able to achieve good health It's so under‐resourced
	Know your community	We are in their environment We've got those cues from the client Back in their environment We have staff that have the personality to take constructive feedback. I think that's a massive part of community [practice] Relying on other people, and relying on networks, and understanding how communities work, understanding family networks in small communities Knowing your community How do you understand your local community and make use of the resources that are available within a local community? The minute you do it the wrong way, particularly Indigenous population, they won't come back and you've lost them
Scope of practice and time	Good triage skills	Time allocation, it's a big part of my triage I don't have a scope of practice and I don't have a real model. I've got to prioritize the most important issue
	Utilise telehealth to save travel time	They got everybody within a couple of suburbs, [and] were then given to one person. It was just actually amazed at how much time was saved in the travel It is sometimes around their internal perception of being strapped for time and not having sufficient time and that actually that perception being a barrier to developing efficient timeliness in practice I think the tele‐health is definitely a standout thing for me there Have ways of tapping into some of the communities that are a little bit more vulnerable by using devices
	Fun in the workplace is important	Having fun in the workplace is an important part of time in the long run Fun in the workplace and being part of their team … you do really often miss out on things
	Community development activities	Doing work in groups, building communities You're building a community to care for themselves Participate in a local community so that you can interact and utilize that relational strength that you have to then refer clients
	Time for paperwork and admin	You admin your time to do your discharges, your time to get your lists and appointments, and all that sort of stuff up to date … I think it takes up more time than when I was using paper It's about all the other stuff that referrals, the other stuff that goes with it but you can't sort of clock up and say “yes well I might have spent 21 min with this woman,” but I spend another hour doing all the behind the scene work for that it's not seen Have to manage a waitlist … whereas allied health are really skilled at doing this … they know how to say no to clinicians, if they've got too many clients/ Time is always an issue Uncaptured workloads … [a task] that's two hours out of your day but it's worth doing and you have to do it
Professional Development & Education	Motivational interviewing	You need somebody good at motivational interviewing to get the information needed out of people in a timely manner Motivational interviewing on the mentorship and support programs Active listening, motivational interviewing, I think is a really good pro community
	Building networks in the community	It's a partnership I see … looking at that you know bigger picture A way we could do a partnership where we may be working with acute facilities How do you build a network in the local community? It's very difficult to work with a multi‐disciplinary team if you don't know each other's role, or that lack of respect, or that lack of knowledge It's unseen work and it's also unseen skill that perhaps isn't recognized in terms of the ability of people to negotiate with stakeholders Being aware of stakeholders and different organizations that are in the community that support clients and families
	Boundaries	Sometimes there are limitations on our practice Remembering what our service was about, what were the core functions of our service … it is so easy to make it bigger, and bigger, and bigger, and then you don't get the time to do the things that really need to be done Because we need to be careful that we're not going beyond what our role is, what our abilities are There's always that reflective question: what am I trained to do? What is in my sphere of influence? I certainly do psychosocial screens. I'm not a social worker, I'm not a counsellor, I'm not a psychologist, that's not my role, I'm not a mental health nurse. So, it's [about] being very firm with other people that they don't expect you to do all those other things that are really outside of what your core business is
	Critical thinking	Critical thinking is an important element Critical thinking because if we can always come back to the play of what does the evidence suggests here Some autonomy in their practice ability to work to the top of their scope They [Generation X and Y] want to be autonomous Be a good problem solver Look at what cannot change
	Wound care and disease specific education	COPDs, your diabetes, your heart disease things … big part of it … wound care Make decisions about what it's going to be the best treatment for this particular wound out in the community
	Transition program with specific CSATs	Disease specific training and skills for hospital avoidance are beneficial We need to have transition to practice programs. We need to have entry levels. We need to start people off and set them up for success, not to make it look too hard and to fail Entry‐level position where it's really clear Ensuring that they have adequate training in all the systems I remember doing some modules on primary health care … there's gaps in that training … not coming from an expert model I've just done a lot of short courses … bought this great book when I nursing for public health
	Health literacy	Someone helping us with reflective practice … we need to always do the evidence‐based practice Having [family] observe [family assessment], and we have video things on that our family partnership process is a framework Understanding what health literacy is … equity and access to services

Each theme comprised of multiple sub‐themes. For example, “safety of self, clinicians and patients” had three sub‐themes: “prevention of hospital admissions,” “social aspects in safety” and “having insight into self and reflective practice.” Each sub‐theme emerged from several participants' statements. For instance, the sub‐theme related to the ability to reflect and have insight into one's practice was supported by the following statements:

you know if you are going home every night wondering if you [have] done the right things … your patient safety… I don't think those feelings ever leave you … professional safety …

need training in this particular area and it's that insight again of knowing where my skills and abilities are…

felt psychologically at risk a number of times in my role in community…

In addition, scope of practice and time was another issue related to safety. “we need to be careful that we're not going beyond what our role is what our abilities are.. to be able to refer on….” This issue emerged under a theme of “professional development and education” and the sub‐theme of boundaries.

“Getting to know your community” was a strong sub‐theme related to attaining a sense of belonging. One participant elaborated: “relying on other people and relying on networks and understanding how communities work understanding family networks in small communities … knowing your community.”

Invisibility of the community nursing was also ubiquitous. The issue of community nursing being poorly understood was evident: “we're not visible they can't see what we're doing so they can't know unless they actually walk in our shoes.” There was also a sense that the specialty is undervalued: “community looks very different to the style of nursing that you see in the acute sector and so being invisible means that we get undervalued.”

The new themes and sub‐themes were converted to quantitative items for round 4 including the new concepts of “conditional requirements,” “for safety” and “orientation requirements” for getting to know the community. Finally, the “specialist workforce retention activity” concept overarched the professional development and lifelong learning of the novice community practice specialist. The findings from the focus group formed the focus of the theme development for round 4.

### 5.4 Round 4

Round 4 consisted of eight closed importance rating questions of 52 items. The results are shown in Tables 12 and 13. Table 12 shows that the highest rating items for the Self fell under Professional—Pre‐entry: “Clinical placement to understand community practice principles and culture” (standardized mean = 4.83); and Personal—Pre‐entry: “Commitment [is] high” (standardized mean = 4.67). Table 13 shows that the highest rating items fell under Strategic Measures (Pre‐entry): “Pre‐entry speciality observation and clinical placement are available” (standardized mean = 4.83); and Transition Processes (Orientation requirements): “Understanding role boundaries within individual scope of practice” (standardized mean = 4.83). The highest rating was for Transitional Processes (Conditional requirement) in the “Ability to provide safe practice in the community setting” (standardized mean = 5.0).

**Table 12:** Round 4 final model ratings for the concept of self

Self	Standardised mean	Median	*SD*
Professional (Pre‐entry) Cronbach's alpha: .960			
Clinical placement to understand community practice principles and culture	4.83	5.00	0.37
Clinical decision‐making developing	4.50	4.50	0.50
Teamwork ability	4.50	4.50	0.50
Professional maturity	4.33	4.50	0.75
Competence, knowledge and skills—generalist	4.33	4.50	0.75
Self‐reflection (i.e. scope of practice)	4.33	4.50	0.75
Critical thinking developing	4.33	4.00	0.47
Leadership skill developing for autonomous practice	4.17	4.00	0.69
Personal preparation through continuous professional development	4.17	4.00	0.37
Future career aspirations in the community speciality	4.17	4.00	0.69
Previous experience, knowledge & skills in the speciality	3.67	4.00	0.47
Personal (Pre‐entry) Cronbach's alpha: .946			
Commitment high	4.67	5.00	0.47
Motivation and passion	4.50	4.50	0.50
Problem‐solving ability is high	4.50	5.00	0.76
Resourcefulness	4.33	4.50	0.75
Coping ability is high	4.33	4.50	0.75
Transition reason positive	4.17	4.00	0.37
Resilience high	4.17	4.00	0.69
Self‐care quality is high	4.17	4.00	0.69
Self‐reflection gives honest personal insight	4.17	4.50	0.90
Self confidence is high	3.67	3.50	0.75

**Table 13:** Round 4 final model ratings for the concepts of strategic measures, transition processes and building credibility

Concept	Standardised mean	Median	*SD*
Strategic Measures (Pre‐entry) Cronbach's alpha: .70			
Pre‐entry speciality observation and clinical placement are available	4.83	5.00	0.37
Transition program is outlined and resourced	4.67	5.00	0.47
Positions in the speciality are available after completing speciality program [i.e. Postgraduate Courses]	4.67	5.00	0.47
Career pathway is defined, outlined and resourced	4.33	4.50	0.75
Speciality is marketed in partnership with education providers such as Universities	4.33	4.00	0.74
Career pathway focus commences at the undergraduate level	4.17	4.50	0.90
Marketing of community nursing career via government and organisation agencies	4.00	4.00	0.58
Transition processes—Orientation requirements Cronbach's alpha: .935			
Understanding role boundaries within individual scope of practice	4.83	5.00	0.41
Buddy system for developing community practice knowledge and skills	4.67	5.00	0.52
Ensure understanding of role in a multidisciplinary service	4.50	4.50	0.55
Knowledge or support systems including peers and other community health professionals	4.33	4.00	0.52
Time frame flexible for orientation tailored to individual practice needs	4.17	4.00	0.75
Transition processes—Support requirements Cronbach's alpha: .906			
Support is provided to develop clinical skills to match community needs	4.83	5.00	0.37
Quality of processes and safety of clients and workforce is a priority	4.83	5.00	0.37
Support is provided for professional well being	4.50	4.50	0.50
Support is provided for continuing education and lifelong learning	4.33	4.00	0.47
Transitional processes—Conditional requirement Cronbach's alpha: .965			
Ability to provide safe practice in the community setting	5.00	5.00	0.00
Ability to have insight into one's individual scope of practice and seek supervision and or referral if needed	5.00	5.00	0.00
Ability to work autonomously	4.50	4.50	0.50
Ability to work in a multidisciplinary team	4.50	4.50	0.50
Person, family and community centre assessment skills are ensured	4.50	4.50	0.50
Knowledge of community culture	4.17	4.50	0.90
Knowledge of referral pathways	3.83	4.00	0.37
Transition process—Specialist workforce retention activity Cronbach's alpha: .988			
The Specialist nurse exhibits outcomes of practice that are professional, capable competent, sustainable and person focused on completion of transition processes	4.67	5.00	0.47
Appropriate skill mix of specialty workforce prevents overwhelming responsibility and workload as the norm	4.67	5.00	0.47
Lifelong learning and reflection are key attributes of the specialist nurse and are supported by the employer	4.67	5.00	0.47
Specialist role is adequately funded post transition processes	4.50	4.50	0.50
Specialty work colleagues respect, include, support and accept the specialist nurse on completion of transition processes	4.50	4.50	0.50
[Positive] culture of the organisation allows development of the professional and personal self	4.50	4.50	0.50
The specialist nurse has a sense of belonging to the community practice	4.50	5.00	0.76
The specialist nurse feels accepted by the community that she/he serves	4.33	4.50	0.75
Building creditability—Education strategy Cronbach's alpha: .940			
Lifelong learning and reflection are key attributes of the specialist nurse and are supported by the employer	4.50	4.50	0.50
Postgraduate community nursing formal education to master level.	4.17	4.00	0.69

**Figure 2:**
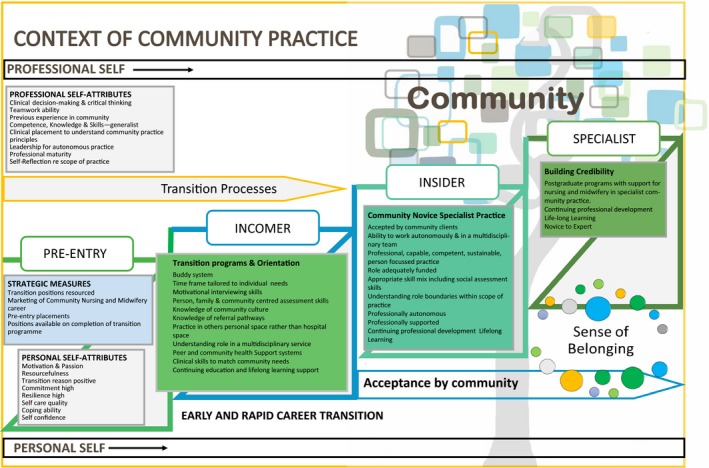
Final model for the early and rapid career transition pathway to specialist community nursing

After the three rounds of Delphi, agreement between panel members remained strong (Tables 7 and 8) with agreement across all items in the questionnaire reaching significance, ICC .964 (95% CI: 0.908–0.994), *F*(5, 255) = 31.332, *p *< .0001. The total Cronbach's Alpha for round 4 model was very strong .964, higher than round 1 and 2 demonstrating strong reliability. All items reached consensus and were included in the final model shown in Figure 2.

### 5.5 The final theoretical model

Figure 2 presents the final model for the early and rapid career transition pathway to specialist community nursing. In the model, the professional and personal Self enter the career pathway at the time point of pre‐entry. This study has addressed the evidence deficiencies in pre‐entry with the addition of pre‐entry strategic measures. Transition programmes and orientation start at pre‐entry and include a formal orientation process that is detailed and specific to the complexities of community practice. As an insider, the pathway continues forward to where acceptance in the community begins, an important concept that underpins retention and a sense of belonging in the context of practice. As the scope of practice does not have pre‐determined professional boundaries or guidelines, it is reliant on self‐regulation to ensure safety for both the specialist nurse and their clients. The personal and professional Self consolidate as Community Novice Specialist Practice evolves from novice to expert through the endeavours of professional and personal development and life‐long learning.

## 6 DISCUSSION

Australia, like many other countries around the world, is facing increasing healthcare pressures. The role of Registered Nurses in community practice and primary healthcare services is central, not only to the delivery of health care, but also to the implementation of health promotion initiatives, preventative strategies and integration of healthcare services (Australian Nursing & Midwifery Federation, 2009). Community and primary health care requires a person‐centred approach and the provision of accessible, essential, integrated and quality care (Stewart et al., 2017).

This Delphi study has provided the knowledge and insight that was deficient in the preliminary model for the early career transition pathway to community nursing. We used the TRANSPEC model of “the effective rapid and early career TRANsition to nursing SPECiality in differing contexts of practice” (Chamberlain et al., 2019). In this study, we found deficiencies in “pre‐entry”—where the marketing of community nursing was negligible and the support around orientation informal and minimal, mainly due to tight budgetary concerns. We found that community practice holds a whole new dimension for nurses transitioning from acute care as the concept of “knowing your community” took time and support. It took time to be accepted in a reciprocal manner between self and the community, and to develop a sense of belonging within the community.

Ashley, Brown, Halcomb, and Peters (2018), in their qualitative work of Registered Nurses transitioning from acute care to primary healthcare employment, dedicate a sizeable proportion of their discussion to orientation during transition. They report similar findings, where orientation was either minimal or non‐existent. The findings emphasize the assumption held by healthcare providers that community nursing is not different to acute hospital nursing, therefore, experienced Registered Nurses transitioning to community practice require minimal orientation. Transition programmes typically include a supernumerary orientation period, structured study days, preceptor or mentor support and access to a nurse educator who usually coordinates the programme (Rush, Adamack, Gordon, Lilly, & Janke, 2013). As highlighted in our study, similar transition to professional practice programmes in community nursing that incorporates a structured orientation in Australia does not widely exist (Murray‐Parahi, DiGiacomo, Jackson, & Davidson, 2016).

“A sense of belonging” to the community relates to the reality that community nursing is grounded in the social model of health. Community nursing practice entails working with individuals, families and community groups (Besner, 2004; Kemp, Anderson, Travaglia, & Harris, 2005). The practice involves coordinating care in multidisciplinary environments and provision of visiting services to clients in complex situations that often require advanced problem‐solving skills (Besner, 2004; Kemp et al., 2005). A “sense of belonging” is an achievement indicating that the community has embraced the practitioner into its privileged state. Attaining a “sense of belonging” is essential not only for optimum practice but also for the long‐term retention of community nurses (Coughlan & Patton, 2018; Moseley, Jeffers, & Paterson, 2008). It also allows for the trust to develop between nurse and client and to establish interagency cooperation for the disclosing of relevant personal and private information (Dellemain, Hodgkin, & Warburton, 2017). Our conceptual model of “the early career transition pathway to specialist community nursing” enhances the TRANSPEC model (Chamberlain et al., 2019; Hegney et al., 2019) and culminates with the novice community nurse specialist at the beginning of the progression from novice to expert (Benner, 2001).

### 6.1 Relevance to clinical practice

This study has identified five key elements needed for effective recruitment, transition and retention of staff in community‐based clinical practice,

1. *Marketing*. There is a general view that community nursing is poorly understood. There is a sense that these professionals are secondary adjuncts to their acute care counterparts, making community nursing an undesirable career pathway. Marketing needs to raise the awareness to the diversity of the role, its autonomy in practice and its generalist–specialist focus. The diverse areas of practice in the community also need to be exposed positively as a professionally satisfying career pathway.
2. *Pre‐entry opportunities need to be provided*. These opportunities should include more clinical placements for nursing students to what are currently available. These placements need to have structured learning activities, provide exposure to clinical activities and be sufficiently long, for students to gain a sound and positive understanding of community practice.
3. *Orientation requirements*. Orientation has been described as ad hoc and not addressing the real needs of a community nurse. Orientation needs to be structured and include key elements of community nursing, rather than be an add‐on to acute based practice. Moreover, orientation should incorporate some introduction to the broader community issues, available support services and referral pathways. For instance, this could include responding to issues such as domestic violence, housing and problematic substance use. In the study, basic operational budgeting was also seen as essential. The implication is that orientation also needs to be flexible enough to recognize the level of experience and practice of Incomers and the application of structured mentoring opportunities that last longer than the orientation timeframes.
4. *Safety for clinicians*. This topic was identified in the Delphi focus group. Concern was raised that there is not enough preparation for dealing with violence in the home, or in related aspects of isolated practice, scope of practice, or the support for rescue and managing such events. Processes and procedures perhaps should be reviewed.
5. *Professional development*. Consistently, reference was made to the difficulties of accessing professional development opportunities, particularly where nurses were in single practice settings. Opportunity to grow and develop critical thinking and a sense of belonging in the community through access to activities in the broader community needs should also be offered. Community‐focused in‐service and tertiary learning opportunities should be developed, in addition to existing specialist‐focused learning.

### 6.2 Limitations

In this study, specific limitations include a low participant number. The panel members were speciality experts and as such their group opinion is considered more “valid” and “reliable” than individual opinion. Although the study was based on the results of a systemic review and its preliminary model, the use of open questions in early rounds of the Delphi opens the study to researcher interpretation, which risks potential bias. To minimize the bias, different members of the research team developed and later analysed open‐ended and closed rating questions. Consensus was achieved by the expert panel. While consensus does not necessarily mean correctness, our model has revealed new knowledge and insights that are grounded in practice. These need to be tested by further research.

## 7 CONCLUSION

This Delphi study presents an emerging early career transition pathway in the speciality of community nursing. The five key elements needed for effective recruitment, transition and retention of staff in community‐based practice included: marketing, formal orientation, personal and professional safety for clinicians and supported professional development. These elements can facilitate effective recruitment, transition and retention of staff in community‐based practice. Future work building and testing this model is a research priority.

## CONFLICT OF INTEREST

No conflicts of interest.

## AUTHOR CONTRIBUTIONS

DC, CH, DH: Conceptualization, formal analysis, methodology, supervision and original draft preparation. DC, CH, DH, LT, AS: Data curation. CH: Funding acquisition. DC, CH, DH, LT, SM, AS, EW, JH, TW: Investigation. DC, CH, TW: Project Administration. CQUniversity: Resources. LT, AS, DC: Validation. DC: Visualization. AS, LT, CH, DH: Review and Editing.

## PATIENT CONSENT STATEMENT

This study did not require a patient consent.

## References

[nop2355-bib-0001] Ashley, C. , Brown, A. , Halcomb, E. , & Peters, K. (2018). Registered nurses transitioning from acute care to primary healthcare employment: A qualitative insight into nurses' experiences. Journal of Clinical Nursing, 27(3–4), 661–668. 10.1111/jocn.13984 28771865

[nop2355-bib-0002] Australian Nursing and Midwifery Federation (2009). Nursing speciality: ANMF policy. Canberra, ACT: ANMF.

[nop2355-bib-0003] Barrett, A. , Terry, D. R. , Lê, Q. , & Hoang, H. (2016). Factors influencing community nursing roles and health service provision in rural areas: A review of literature. Contemporary Nurse, 52(1), 119–135. 10.1080/10376178.2016.1198234 27264878

[nop2355-bib-0004] Benner, P. (2001). From novice to expert: Excellence and power in clinical nursing practice (Commemorative ed.). Upper Saddle River, New Jersey: Prentice‐Hall.

[nop2355-bib-0005] Besner, J. (2004). Nurses’ role in advancing primary health care: A call to action. Primary Health Care Research & Development, 5(4), 351–358. 10.1191/1463423604pc225oa

[nop2355-bib-0006] Burnard, P. (1991). A method of analysing interview transcripts in qualitative research. Nurse Education Today, 11(6), 461–466. 10.1016/0260-6917(91)90009-Y 1775125

[nop2355-bib-0035] Castaneda, G. A. , & Scanlan, J. M. (2014). Job satisfaction in nursing: A concept analysis. Nursing Forum, 49(2), 130–138. 10.1111/nuf.12056 24383666

[nop2355-bib-0007] Chamberlain, D. , Hegney, D. , Harvey, C. , Knight, B. , & Tsai, L. (2019). The factors influencing the effective early career and rapid transition to a nursing specialty in differing contexts of practice: A modified Delphi consensus study. British Medical Journal Open, Submitted May 2019. (in press) 10.1136/bmjopen-2018-028541 PMC6720241 31462470

[nop2355-bib-0038] COAG Health Council (2014). Guidance for National Board Submission re Specialist Registration.

[nop2355-bib-0008] Coughlan, L. M. , & Patton, D. (2018). A qualitative descriptive exploration of the educational and career plans of early career neonatal nurses and midwives: An Irish perspective. Nurse Education in Practice, 28, 182–188. 10.1016/j.nepr.2017.10.026 29102854

[nop2355-bib-0009] de Loë, R. C. , Melnychuk, N. , Murray, D. , & Plummer, R. (2016). Advancing the state of policy Delphi practice: A systematic review evaluating methodological evolution, innovation and opportunities. Technological Forecasting and Social Change, 104, 78–88. 10.1016/j.techfore.2015.12.009

[nop2355-bib-0010] Dellemain, J. , Hodgkin, S. , & Warburton, J. (2017). Time, terrain and trust: Impacts of rurality on case management in rural Australia. Journal of Rural Studies, 49, 50–57. 10.1016/j.jrurstud.2016.11.006

[nop2355-bib-0011] Greatorex, J. , & Dexter, T. (2000). An accessible analytical approach for investigating what happens between the rounds of a Delphi study. Journal of Advanced Nursing, 32(4), 1016–1024. 11095243

[nop2355-bib-0012] Harvey, C. , Hegney, D. , Sobolewska, A. , Chamberlain, D. , Wood, E. , Wirihana, L. , … Wake, T. (2019). Developing a community‐based nursing and midwifery career pathway–A narrative systematic review. PLoS ONE, 14(3), e0211160 10.1371/journal.pone.0211160 30921338 PMC6438448

[nop2355-bib-0013] Harvey, C. , Hegney, D. , Tsai, L. , Mclellan, S. , Chamberlain, D. , Sobolewska, A. , … Wake, T. (2019). Nurses’ experiences of transition to community‐based practice. Clinical Nursing Studies, 7(3), 1–11. 10.5430/cns.v7n3p1.

[nop2355-bib-0014] Hegney, D. , Chamberlain, D. , Harvey, C. , Sobolewska, A. , Knight, B. , & Garrahy, A. (2019). From incomer to insider: The development of the TRANSPEC model–A systematic review of the factors influencing the effective rapid and early career TRANsition to a nursing SPECiality in differing contexts of practice. PLoS ONE, 14(5), e0216121 10.1371/journal.pone.0216121 31042747 PMC6494050

[nop2355-bib-0015] Heiko, A. (2012). Consensus measurement in Delphi studies: Review and implications for future quality assurance. Technological Forecasting and Social Change, 79(8), 1525–1536.

[nop2355-bib-0016] Humphreys, J. , Wakerman, J. , Kuipers, P. , Russell, D. , Siegloff, S. , Homer, K. , & Wells, R. (2017). Improving workforce retention: Developing an integrated logic model to maximise sustainability of small rural and remote health care services. Canberra, ACT: Australian Primary Health Care Research Institute.

[nop2355-bib-0017] Keeney, S. , Hasson, F. , & McKenna, H. P. (2001). A critical review of the Delphi technique as a research methodology for nursing. International Journal of Nursing Studies, 38(2), 195–200. 10.1016/S0020-7489(00)00044-4 11223060

[nop2355-bib-0018] Kemp, L. , Anderson, T. , Travaglia, J. , & Harris, E. (2005). Sustained nurse home visiting in early childhood: Exploring Australian nursing competencies. Public Health Nursing, 22(3), 254–259. 10.1111/j.0737-1209.2005.220309.x 15982199

[nop2355-bib-0019] Kingma, M. (2018). Nurses on the move: Migration and the global health care economy. London, UK: Cornell University Press.

[nop2355-bib-0020] Marć, M. , Bartosiewicz, A. , Burzyńska, J. , Chmiel, Z. , & Januszewicz, P. (2018). A nursing shortage–a prospect of global and local policies. International Nursing Review, 16(1), 9–16. 10.1111/inr.12473 30039849

[nop2355-bib-0021] Mitroff, I. , & Turoff, M. (2002). Philosophical and methodological foundations of Delphi In Linstone M. H. T. (Ed.), The Delphi method: Techniques and applications (pp. 17–34). Neward, NJ: New Jersey Institute of Technology.

[nop2355-bib-0022] Moloney, W. , Boxall, P. , Parsons, M. , & Cheung, G. (2018). Factors predicting Registered Nurses’ intentions to leave their organization and profession: A job demands‐resources framework. Journal of Advanced Nursing, 74(4), 864–875. 10.1111/jan.13497 29117451

[nop2355-bib-0023] Moseley, A. , Jeffers, L. , & Paterson, J. (2008). The retention of the older nursing workforce: A literature review exploring factors that influence the retention and turnover of older nurses. Contemporary Nurse, 30(1), 46–56. 10.5172/conu.673.30.1.46 19072190

[nop2355-bib-0024] Murray‐Parahi, P. , DiGiacomo, M. , Jackson, D. , & Davidson, P. M. (2016). New graduate registered nurse transition into primary health care roles: An integrative literature review. Journal of Clinical Nursing, 25(21–22), 3084–3101. 10.1111/jocn.13297 27350660

[nop2355-bib-0037] National Nursing Organisations (2004). Criteria for Specialities in Nursing/Principles of Credentialling for Nurses. Kingston, NY: National Nursing Organisations.

[nop2355-bib-0025] Norman, G. (2010). Likert scales, levels of measurement and the “laws” of statistics. Advances in Health Sciences Education, 15(5), 625–632. 10.1007/s10459-010-9222-y 20146096

[nop2355-bib-0039] Nursing and Midwifery Board of Australia (2018). Code of Conduct 2.2. Nursing and Midwifery Board of Australia.

[nop2355-bib-0026] Pearson, A. , Hegney, D. , Donnelly, P. (2000). Serving the community: The rural general In Gott M. (Ed.), Nursing practice, policy and change (pp. 143–160). Boca Raton, FL: CRC Press.

[nop2355-bib-0036] Queensland Health (2016). My health, Queensland's future: Advancing health 2026. Brisbane, Australia: Queensland Govt Retrieved from www.health.qld.gov.au/system-governance/strategic-direction/plans/vision-strategy/

[nop2355-bib-0027] Rayens, M. K. , & Hahn, E. J. (2000). Building consensus using the policy Delphi method. Policy, Politics, & Nursing Practice, 1(4), 308–315. 10.1177/152715440000100409

[nop2355-bib-0028] Rush, K. , Adamack, M. , Gordon, J. , Lilly, M. , & Janke, R. (2013). Best practices of formal new graduate nurse transition programs: An integrative review. International Journal of Nursing Studies, 50(3), 345–356. 10.1016/j.ijnurstu.2012.06.009 22795800

[nop2355-bib-0029] Smith, J. A. , & Herriot, M. (2017). Positioning health promotion as a policy priority in Australia. Health Promotion Journal of Australia, 28(1), 5–7. 10.1071/HEv28n1_ED2 29248042

[nop2355-bib-0030] Stewart, G. , Bradd, P. , Bruce, T. , Chapman, T. , McDougall, B. , Shaw, D. , & Soars, L. (2017). Integrated care in practice–the South Eastern Sydney experience. Journal of Integrated Care, 25(1), 49–60. 10.1108/JICA-07-2016-0025

[nop2355-bib-0031] Tavakol, M. , & Dennick, R. (2011). Making sense of Cronbach's alpha. International Journal of Medical Education, 2, 53 10.5116/ijme.4dfb.8dfd 28029643 PMC4205511

[nop2355-bib-0032] Turoff, M. (1970). The design of a policy Delphi. Technological Forecasting and Social Change, 2(2), 149–171. 10.1016/0040-1625(70)90161-7

[nop2355-bib-0033] Twigg, D. , & McCullough, K. (2014). Nurse retention: A review of strategies to create and enhance positive practice environments in clinical settings. International Journal of Nursing Studies, 51(1), 85–92. 10.1016/j.ijnurstu.2013.05.015 23809644

[nop2355-bib-0034] World Health Organization (2015). WHO global strategy on people‐centred and integrated health services: Interim report. Geneva, Switzerland: WHO.

